# Exploring Pain and Body Composition in Children with Cancer Compared to Healthy Controls: A Cross-Sectional Case-Control Study

**DOI:** 10.3390/children12091166

**Published:** 2025-09-01

**Authors:** Sophie Pleysier, Kelly Ickmans, Anneleen Malfliet, Aline Wauters, Jutte van der Werff ten Bossch, Sara Debulpaep, Amelien Vanacker, Tine Vervoort, Perseverence Savieri, Emma Rheel

**Affiliations:** 1 Pain in Motion Research Group (PAIN), Department of Physiotherapy, Human Physiology and Anatomy, Faculty of Physical Education & Physiotherapy, Vrije Universiteit Brussel, 1090 Brussels, Belgium; sophie.pleysier@vub.be (S.P.); kelly.ickmans@vub.be (K.I.); anneleen.malfliet@vub.be (A.M.); 2 Department of Physical Medicine and Physiotherapy, University Hospital Brussels, 1090 Brussels, Belgium; 3 Research Foundation—Flanders (FWO), 1000 Brussels, Belgium; 4 Department of Experimental-Clinical and Health Psychology, Ghent University, 9000 Ghent, Belgium; aline.wauters@ugent.be (A.W.); tine.vervoort@ugent.be (T.V.); 5 Department of Pediatrics, University Hospital Brussels, 1090 Brussels, Belgium; jutte.vanderwerfftenbosch@zas.be; 6 Department of Pediatrics, Ghent University Hospital, 9000 Ghent, Belgium; sara.debulpaep@uzgent.be (S.D.);; 7 Core Facility—Support for Quantitative and Qualitative Research (SQUARE), Vrije Universiteit Brussel, 1090 Brussels, Belgium; perseverence.savieri@vub.be; 8 Biostatistics and Medical Informatics Research Group, Vrije Universiteit Brussel, 1090 Brussels, Belgium

**Keywords:** children, cancer, pain, pressure pain thresholds, body composition, case-control

## Abstract

**Highlights:**

This article presents the results of a cross-sectional study exploring differences and associations in pain and body composition in children with cancer compared to healthy controls.

**What are the main findings?**

- Children with cancer showed differences in both pain outcomes (i.e., higher chronic pain prevalence, lower pressure pain thresholds) and body composition (i.e., higher waist circumference and fat percentage, and lower body water percentage) compared to age- and sex-matched healthy peers.
- Pressure pain thresholds were associated with cancer diagnosis and lower muscle mass, suggesting a link between pain and body composition.

**What is the implication of the main finding?**

- The findings may guide clinical conversations on pain management and body composition monitoring in pediatric cancer care.

**Abstract:**

**Background:** Children with cancer frequently experience pain, which may persist into survivorship. Furthermore, many undergo body composition changes throughout their disease trajectory. However, little is still known about the interplay between pain and body composition. **Methods:** This cross-sectional case–control study compared pain and anthropometric characteristics between 30 children with cancer (8–18 years) and 30 age- and sex-matched healthy controls and examined whether pain was associated with anthropometric characteristics and cancer diagnosis. Pain in the past two weeks, chronic pain, and pressure pain thresholds (PPTs) at the tibialis anterior and trapezius pars descendens muscles were assessed. Anthropometric measures included waist circumference, fat %, fat-free mass, muscle mass, body water %, and Body Mass Index. **Results**: Children with cancer had a higher prevalence of chronic pain (*p* = 0.011), lower PPTs at the tibialis anterior (*p* = 0.030), and fewer pain locations (*p* = 0.037). They also showed lower body water % (*p* = 0.020), and higher waist circumference (*p* = 0.012) and fat % (*p* = 0.026). Cancer diagnosis and lower muscle mass were associated with lower PPTs at both locations (tibialis anterior: *p* = 0.016, β = −0.305; *p* = 0.033, β = 0.267; trapezius pars descendens: *p* = 0.020, β = −0.286; *p* = 0.004, β = 0.361, respectively). **Conclusions:** Children with cancer differ from their healthy peers in both pain and body composition profiles. These findings underscore the need for systematic pain assessment and body composition monitoring in pediatric oncology and may help identify children at risk for heightened pain sensitivity and adverse body composition changes who could benefit from early, targeted interventions.

## 1. Introduction

Each year, globally, about 400,000 children and adolescents (0–19 yo) are diagnosed with cancer [1]. Throughout the disease trajectory they are frequently confronted with pain (28–62%) [2,3,4,5,6,7,8], which may persist into survivorship [9,10,11]. Various factors may underlie cancer-related pain, such as medical treatments (e.g., chemotherapy, radiation, surgery), treatment-related procedures (e.g., bone marrow aspirations, catheter access, lumbar punctures), and the disease itself (e.g., tumor infiltration) [10,12,13,14]. Treatment-related pain is most prevalent [11,15,16], with chemotherapy-induced neuropathic pain most frequently reported [17,18,19]. Pain due to treatment-related procedures is very common as well and often provokes significant anxiety and distress [20,21]. Moreover, cancer-related pain may persist and negatively impact the child’s life in the long term. Indeed, repeated exposure to invasive medical procedures may increase predisposition to chronic pain in adulthood [22]. These long-term consequences may be explained by central nervous system alterations (i.e., peripheral or central hyperexcitability), causing other spontaneous pains and skin allodynia or hyperalgesia associated with reduced pain thresholds [23,24].

Further, many children with cancer experience changes in body composition, which can already be observed early in treatment [25]. While the reported changes vary, results overall indicate increased fat mass, decreased body cell mass, and decreased fat-free mass, either with or without increased body weight or body mass index (BMI) [25,26,27]. These alterations may result in overweight-related complications (e.g., higher risk for relapse and lower survival rates [27,28,29,30,31,32]), and might be associated with (chronic) pain [33]. Potential mechanisms include treatment-induced endocrine disruption and altered metabolism [34], systemic inflammation and muscle wasting [35,36,37], and reduced physical activity during and after treatment [38,39]. In turn, excess adiposity and reduced muscle mass may sensitize nociceptive pathways and promote pro-inflammatory cytokine release [40], thereby increasing pain vulnerability. Conversely, persistent pain may reduce mobility and physical activity, further exacerbating body composition changes [41]. This bidirectional relationship highlights the importance of investigating both pain and body composition, as well as their interplay, in pediatric oncology.

Despite evidence that children with cancer frequently experience pain and undergo body composition changes, few studies have examined these two factors together, and the potential interplay between pain sensitivity and body composition remains poorly understood in this population. Existing research has mainly described prevalence rates of pain or reported treatment-related changes in weight and body composition but has not systematically investigated their associations or compared them with healthy peers. This gap is clinically relevant, as both pain and adverse body composition may persist into survivorship and contribute to long-term health risks. To address this gap, this exploratory cross-sectional case–control study examined differences in pain (i.e., pain experience, pressure pain thresholds (PPTs)) and anthropometric characteristics (i.e., waist circumference, BMI, body composition) in children with cancer (8–18 yo) compared to age- and sex-matched healthy controls. Additionally, the study explored associations between pain, anthropometric characteristics and cancer diagnosis. The findings can enhance understanding of the effects of cancer and its treatment and inform on the most appropriate pain management approaches.

## 2. Materials and Methods

### 2.1. Setting and Participants

Ethical approval for this cross-sectional case–control study was obtained from the Ethics Committee of the Vrije Universiteit Brussel/University Hospital Brussels and Ghent University/University Hospital Ghent in February 2019 and was registered at clinicaltrials.gov (NCT04004455) in June 2019. Informed consent forms were proofread by 2 children from the relevant age range. Participants included children (8–18 yo) undergoing active cancer treatment at the pediatric Hemato-Oncology and Stem Cell Transplantation department of the University Hospital Brussels or University Hospital Ghent, and age- and sex-matched healthy controls recruited from nearby schools and the researchers’ network. Study recruitment and participation occurred between July 2019 and May 2022. Written informed consent/assent was obtained from both the child and the participating parent before study participation.

Exclusion criteria for participation were (1) developmental disabilities (e.g., autism spectrum disorder, attention deficit (hyperactivity) disorder, developmental coordination disorder), (2) mental impairment or psychiatric disorders (e.g., anxiety disorder, personality disorder), significant vision or hearing impairment, (3) underlying primary chronic pain disorders (e.g., fibromyalgia, migraine, juvenile idiopathic arthritis), (4) cancer relapse (i.e., not first cancer diagnosis), and (5) insufficient Dutch language proficiency. The lower age limit (i.e., 8 yo) was based on the used self-report questionnaires [42]. A priori sample size calculation (G*Power 3.1.9.2; Franz Faul, Kiel, Germany) was based on a study examining pain hypersensitivity in children with juvenile idiopathic arthritis compared with healthy controls [43], and estimated a sample size of 26 participants per group for 80% study power (α = 0.05, N1/N2 = 1). Accounting for a 10% dropout, the aim was to include at least 30 participants per group, resulting in a total of 60 participants.

### 2.2. Study Flow

Initial screening of children with cancer was based on age, sex, and other medical or developmental disorders mentioned in their medical records. Eligible children and their parents received standardized study information from the research team during a hospital visit. Upon obtaining written informed consent/assent, study participation took place during the next hospital visit in the child’s hospital room or an empty room at the department. Healthy children were recruited via social media and flyers. Interested families received standardized study information via email and, when provided written informed consent/assent, both child and parent were invited to the laboratory at Ghent University or Vrije Universiteit Brussel for study participation. First, both child and parent independently completed online socio-demographic and pain-related questionnaires (see ‘Measurements’). Next, children’s body length, waist circumference, body composition, and PPTs were measured, in respective order.

### 2.3. Measurements

#### 2.3.1. Socio-Demographic Characteristics

Children reported their biological sex and age. Parents of children with cancer also reported on their child’s medical information (i.e., diagnosis, date of diagnosis, medication), confirming information from their medical record. All parents reported on socio-demographic variables, including biological sex, age, health status, family situation, educational level, and occupation.

#### 2.3.2. Pain Characteristics

*Pain experience*—Children reported their pain experience in the past 2 weeks (yes/no). Those answering “yes” rated their pain frequency and intensity on separate 4-point scales and marked their pain location(s) on a body chart with 21 predefined areas. The total number of locations marked by each individual was used for statistical analysis. Children who answered “no” were assigned a score of 0 for pain frequency, pain intensity, and number of pain locations over the past 2 weeks. In addition, all parents, regardless of their child’s response to pain experience in the past 2 weeks, indicated whether their child had chronic pain (i.e., pain persisting or recurring for >3 months [44], yes/no).

*Pressure algometry—Pressure Pain Thresholds* (PPTs) were measured at the dominant musculus tibialis anterior (MTA) and musculus trapezius descendens (MTD), using a digital pressure algometer with a 1 cm^2^ rubber probe (FPX 50, Wagner Instruments, Greenwich, CT, USA) [45,46]. PPTs were measured at two muscle sites (MTA and MTD) in line with pediatric pain research guidelines, which recommend limiting the number of test locations to reduce participant burden, distress, and potential variability while still allowing assessment of both localized and generalized pain sensitivity [47]. The children were asked to sit on a chair, with their arms comfortably resting on the chair grips and their feet fully supported on the ground (or a step stool for the smallest children). Hips, knees and feet were positioned at a 90° angle. The algometer was placed perpendicular to the skin’s surface, and the pressure was manually increased based on prior training [48]. The investigator was blinded to the applied pressure to the extent possible, by turning the screen of the algometer downwards. The children were instructed to say “stop” as soon as the pressure became ‘uncomfortable’ [45,48]. The sequence of the test sites was randomized by having the child choose between 2 cards lying upside down on the table. The muscle written on the reverse side of the chosen card (i.e., MTA or MTD) served as the first test location. A total of 3 measurements were performed at both locations, with 30 s between each measurement [49]. The mean peak kg/cm^2^ value of the final 2 measurements was considered as the location-specific PPT [48,49,50], with higher values indicating lower pressure pain sensitivity. All participants were blinded from the applied pressure to their skin.

#### 2.3.3. Anthropometric Characteristics

Before anthropometric measurements, participants were asked to go to the toilet (to empty their bladder) and remove their clothes, except for their underwear.

*Body Height*—Children’s body height was assessed using a Seca 2013 mobile Stadiometer (Seca Benelux, Gooimeer, the Netherlands), positioned on a flat surface. The children were positioned centrally in front of the stadiometer, with their ankles at a 90° angle and their heels together, against the base of the stadiometer. Children were asked to make themselves as long as possible, without lifting their heels. Heels, calves, buttocks and shoulders were positioned along an imaginary vertical line. The children’s heads were tilted to keep the ear canal and the orbit in an imaginary horizontal line, perpendicularly oriented to the stadiometer. The Stadiometer’s headpiece was lowered until it touched the head and slightly compressed the hair. The body height was noted to the nearest 0.10 cm, and used for further measurements (see ‘*Body composition*’).

*Waist circumference*—Children’s waist circumference was assessed using a circumference tape (Seca 201 length meter; Seca Benelux, Gooimeer, the Netherlands), positioned at 4 cm above the umbilicus, in a standing position, arms hanging relaxed along the body [51]. The lower edge of the tape was placed onto the skin, parallel to the transverse plane and perpendicularly to the longitudinal axis of the body [52]. The children were instructed to breathe calmly and not to tuck in their bellies during the measurements. The waist circumference at the end of the children’s third expiration was noted to the nearest 0.10 cm.

*Body composition*—Body fat percentage (%), fat-free mass (kg), muscle mass (kg), body water percentage (%), and BMI (kg/m^2^) were measured simultaneously using a TANITA body composition analyzer (TANITA MC-780 SMA from Tanita Corporation; Tanita Europe, Hoogoorddreef, the Netherlands), which operates based on bioelectrical impedance. Prior to measurement, the children’s biological sex, age and body height were entered. Afterwards, the children were asked to stand barefoot on the TANITA scale, with toes and heels in contact with the anterior and posterior electrodes on the platform, respectively. The body composition analysis started when the children were asked to grasp the TANITA grips with both hands. The built-in equation converted the input impedance to body composition estimates.

### 2.4. Statistical Analyses

Analyses were conducted using IBM SPSS Statistics 29 (SPSS IBM, New York City, NY, USA). Descriptive characteristics were reported as means (standard deviations) for continuous and frequencies (%) for categorical data. Group differences were analyzed using independent *t*-tests for continuous normal distributed variables, Mann–Whitney U tests for continuous non-normal distributed data, and chi-square/Fisher’s Exact tests for categorical data. Data distribution was assessed via histograms, QQ-plots, and the Shapiro–Wilk test. Regression analyses examined associations between group (children with cancer vs. healthy controls) and anthropometric characteristics (fat %, muscle mass, BMI) as independent variables, and pain characteristics (PPTs at the MTA and MTD, and number of pain locations in the past 2 weeks) as outcomes. Multicollinearity was checked using a correlation matrix and Variance Inflation Factor. Age and sex were not considered for the regression analyses as participants were matched on these variables, and no variability was expected. A negative binomial regression analysis was conducted for the number of painful locations, using a log link function. Model fit and model comparison were assessed using deviance and Pearson chi-square values, and Akaike Information Criterion (AIC) and Bayesian Information Criterion (BIC). Multiple regression analyses identified variables most strongly associated with PPTs at both muscle sites, using stepwise entry in the model of the MTA and simultaneous entry in the model of the MTD. The data for PPT at the MTD were log-transformed since the assumption of normally distributed residuals was violated in the initial model. Model selection was performed using AIC and BIC. Results were reported as regression coefficients (B) and *p*-values, with statistical significance set at *p* < 0.05.

## 3. Results

### 3.1. Participants

In total, 42 children with cancer from the University Hospital of Ghent and Brussels and their parents were informed about the study during a routine hospital appointment. Five children were excluded from participation because their parents had a poor understanding of the Dutch language (n = 2), the child had attention deficit hyperactivity disorder and anxiety disorder (n = 1), stopped treatment (n = 1), or was <8 yo (n = 1). Seven children declined to participate due to being too distressed (n = 4), feeling too sick (n = 1), or unspecified reasons (n = 2). Among 48 interested healthy controls and their parents, 18 child–parent dyads were excluded due to non-response to the email with study information and subsequent reminder (n = 5), lack of time (n = 6), child’s refusal (n = 1), or the child had a poor health condition (n = 2), autism spectrum disorder (n = 2), developmental coordination disorder (n = 1), or severe dyslexia (n = 1). The final sample consisted of 60 children (8–18 yo), including 30 children with cancer and 30 healthy controls, each group comprising 13 girls and 17 boys. Descriptive characteristics for both groups separately are presented in Table 1. Medical characteristics of children with cancer are presented in Table 2.

### 3.2. Differences in Pain Characteristics

**Figure 1:**
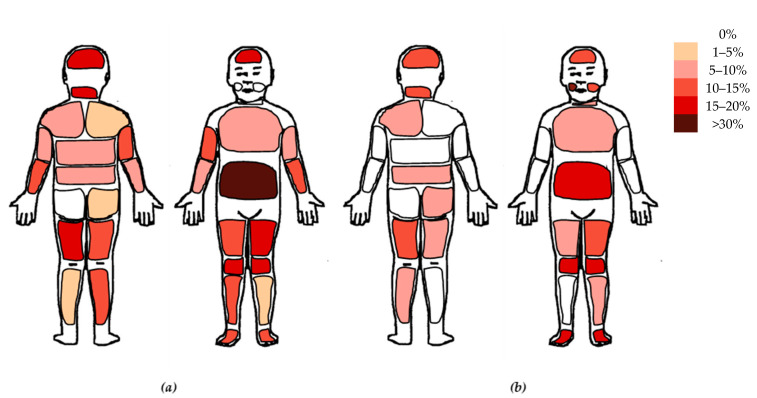
Heat map of painful locations in children who reported to have experienced pain in the past 2 weeks: (**a**) painful locations reported by healthy controls (*n* = 23); (**b**) painful locations reported by children with cancer (*n* = 16). Darker shades indicate higher percentages (range: 0% = white, >30% = dark brown). Exact percentages per body location are available in Supplementary Table S1.

Children’s pain-related characteristics for both groups separately are presented in Table 3. Between-group analyses indicated that significantly more children with cancer had chronic pain compared to their healthy peers (*p* = 0.011). None of the healthy controls had chronic pain. Of the 7 children with cancer with chronic pain, 2 children were diagnosed <3 months before participation and were reporting burning neck pain and headache, respectively, for >6 months. The other 5 children experienced chronic or recurrent headache (n = 1), stomach ache (n = 2), musculoskeletal pain (n = 1), and widespread pain including pain in the jaws (n = 1) since treatment onset. Further, children with cancer showed significantly lower PPTs at the MTA (*p* = 0.030) and fewer pain locations in the past 2 weeks (*p* = 0.037). No other significant between-group differences were found. Pain locations reported by children experiencing pain in the past 2 weeks are visualized using heat maps (Figure 1a: healthy controls; Figure 1b: children with cancer), with darker shades indicating a higher percentage of children who indicated the location as painful. Supplementary Table S1 provides the exact percentages per location for both groups. Overall, quite similar reported pain locations are observed; however, the jaws were not indicated by healthy controls, while 3 children with cancer reported pain in the left and/or right jaw. Additionally, some regions (e.g., the arms, upper back, and right lower leg) were indicated as painful by >5% of healthy controls, but not by children with cancer. In children with cancer the most common pain locations were the jaws, abdomen, knee and feet.

### 3.3. Differences in Anthropometric Characteristics

Children’s anthropometric characteristics for both groups separately are presented in Table 4. Analysis of between-group differences indicated that waist circumference (*p =* 0.012) and fat % (*p* = 0.026) were significantly higher, and total body water % (*p* = 0.020) was significantly lower in children with cancer compared to their healthy peers. No other significant between-group differences were found.

### 3.4. Associations Between Cancer Diagnosis, Anthropometric Characteristics, and Pain Characteristics

Associations between group (i.e., children with cancer versus healthy controls), anthropometric variables (i.e., fat %, muscle mass, BMI), and pain characteristics (i.e., the number of painful locations in the past 2 weeks, and PPTs at the MTA and MTD) can be found in Table 5.

The negative binomial regression model for the number of painful locations was statistically significant, indicating that the independent variables, as a set, were related to the number of painful locations experienced over the past 2 weeks (*p* = 0.024). No significant associations were found for cancer diagnosis or anthropometric variables, in relation to the number of painful locations.

The final multiple regression models for the PPT at the MTA (*p* = 0.009) and the log-transformed PPT at the MTD (*p* = 0.002) were statistically significant, explaining 15.1% and 19.5% of the variance in PPTs respectively. Cancer diagnosis was significantly related to PTTs, indicating that children with cancer tend to have lower PPTs (i.e., higher pressure pain sensitivity) at both muscle sites compared to their healthy peers, controlling for muscle mass (B = −1.04, *p* = 0.016 for MTA, and B = −0.37, *p* = 0.020 for MTD). Additionally, a positive association was found for muscle mass, suggesting that higher muscle mass was associated with higher PPTs (i.e., lower pressure pain sensitivity) across both muscle sites, controlling for the presence of a cancer diagnosis (B = 0.05, *p* = 0.033 for MTA, and B = 0.03, *p* = 0.004). No other significant associations were identified.

## 4. Discussion

This study examined differences in pain and anthropometric characteristics between children with cancer and age- and sex-matched healthy controls and explored associations between these outcomes. Several group differences were found, and both cancer diagnosis and anthropometric characteristics showed associations with pain characteristics.

### 4.1. Differences in Pain Characteristics

Study findings indicated that 23.33% of children with cancer experienced chronic pain, consistent with the current literature on pain prevalence during active cancer treatment varying from 28–62% [2,3,4,5,6,7,8,53]. In contrast, none of the healthy controls reported chronic pain, despite literature showing that chronic and recurrent pain affects up to 21% of children and adolescents [54]. Potential explanations for this unexpected finding include children experiencing chronic pain not feeling encouraged to participate as ‘healthy’ controls, exclusion of children with primary pain disorders, and parent-reported pain assessment underestimating their child’s pain duration. The most commonly reported pain locations in children with cancer were the jaws, abdomen, knees and feet, consistent with previous studies [53,55]. However, to date, the difference in the number of pain locations between children with cancer and healthy controls remained unexplored. Surprisingly, in the present study children with cancer reported fewer pain locations. Since pain in children with cancer is often treatment-induced [11,15,16], these children may be more focused on reporting treatment-related pain, potentially overlooking other locations.

In addition, PPTs measured at the MTA (but not at the MTD) were lower in children with cancer, indicating higher pressure pain sensitivity. Few previous studies have measured PPTs in childhood cancer survivors, of which 2 studies [56,57] reported pathologic sensitization including pressure hyperalgesia in 41% [56] and >50% [57] as part of a more comprehensive Quantitative Sensory Testing (QST) protocol. Another study [58] found that 86% of childhood cancer survivors showed at least 1 abnormal QST parameter, although PPTs were not assessed. The current findings in children undergoing treatment align with these findings in childhood cancer survivors. Importantly, both the small median difference (pain locations) and effect size (both pain locations and PPT MTA) suggest limited clinical relevance, despite reaching statistical significance.

Underlying mechanisms contributing to altered pain characteristics in children with cancer are likely multifactorial, including treatment-related pain, procedural pain, and pain due to the disease itself [10,12,13,14,19]. Inflammatory processes and cytokine release during treatment can enhance peripheral sensitization, while repeated painful procedures and ongoing nociceptive input may promote central sensitization [58], as suggested by the observed lower PPTs. In addition, psychosocial factors such as anxiety and heightened attention to treatment-related pain may further influence pain perception [59,60]. Together, these mechanisms may help explain both the presence of chronic pain and the increased pressure pain sensitivity observed in children with cancer.

### 4.2. Differences in Anthropometric Characteristics

Children with cancer had a higher fat % compared to healthy peers. These findings are consistent with previous findings of increased body fat % and trunk fat which can already be observed in the first months of cancer treatment [25,27,61]. Compared with age- and sex-specific body fat reference curves [62], 90% of healthy controls had a normal body fat %, while 66.67% of children with cancer fell within the normal range, and a minority would be classified as underfat (3.33%), overfat (20%), or obese (10%). Despite the majority falling within a normal body fat range, adequately monitoring body composition in childhood cancer remains crucial, as treatment-related fat gain can persist into survivorship [27,31,61,63] and increase the risk of future comorbidities [28,29,64].

In addition to a higher fat %, children with cancer showed a higher waist circumference, which is an important predictor of central obesity and an essential criterion to define metabolic syndrome [65,66]. Compared to reference values from healthy Belgian youth [67], 1 healthy child (3.33%), and 8 children (27.59%) with cancer exceeded the recommended cut-off, suggesting a trend toward central obesity and metabolic syndrome in children with cancer [66]. However, these findings should be interpreted with caution, as actual risk estimation requires additional factors [66]. Moreover, methodological differences in waist circumference measurement should be considered when comparing the current results to these reference values.

Contrary to the expectation of corticosteroid-related fluid retention [68,69], children with cancer showed a lower total body water %. However, this aligns with the observed higher fat %, since fatty tissue contains less water compared to lean tissue (key principle of bioelectrical impedance analysis). In both groups, the majority had a total body water % within the healthy range [70,71].

It is again important to note when interpreting these results that, for body fat % and total body water %, the small effect size suggests limited clinical relevance, despite reaching statistical significance.

Regarding body weight and BMI in children with cancer, studies show mixed results, with some indicating an increase during and even after treatment [26,61,63], while others report no differences [25,27]. The present findings align with the latter, showing no differences in BMI between children with cancer and healthy peers. In studies reporting no changes, these findings could be explained by opposing changes in fat and lean body mass resulting in a break-even body weight and BMI [25,72]. This study indeed demonstrated a higher body fat % in children with cancer; however, no difference in fat-free mass was observed. Furthermore, according to the World Health Organization BMI-for-age reference data (5–19 yo), 4 children with cancer would be classified as overweight (13%), 1 as obese (3%), and 2 as underweight (7%) [73]. Although the majority (77%) had a BMI within the healthy range, monitoring throughout treatment remains crucial, including in those identified with initially normal or low BMI values, to prevent weight and fat gain post-treatment [63].

### 4.3. Associations Between Cancer Diagnosis, Anthropometric Characteristics, and Pain Characteristics

Children with cancer tend to have lower PPTs at both muscle sites, which strengthens the hypothesis of a different pain profile compared to healthy children. As this relationship has not been directly examined before, further research is needed to confirm and clarify these associations.

Muscle mass was positively associated with PPTs at both sites, suggesting that children with higher muscle mass are less sensitive to pressure pain. However, the overall variance could only be explained for 15.1% for the MTA and 19.5% for the MTD, indicating a limited but potentially protective role for muscle mass in pain sensitivity. Comparison with existing literature is challenging due to methodological differences. Ferreira et al. [74] found that lower skeletal muscle mass was associated with higher pressure hyperalgesia at the MTA in young adults with patellofemoral pain, possibly due to more efficient conditioned pain modulation, an indicator of endogenous pain inhibition [75]. They also found higher body fat to be associated with higher pressure hyperalgesia [74]. As underlying mechanism they hypothesized that proinflammatory cytokines produced by fat tissue could sensitize peripheral nociceptors and central nociceptive transmission pathways, contributing to increased pain sensitivity [74,76,77]. Interestingly, the present study results revealed no association between fat % and PPTs, suggesting a more nuanced role in pain perception.

BMI was not significantly associated with either the number of pain locations or PPTs, consistent with previous research [74]. BMI may be too general as anthropometric measure to accurately assess associations with specific pain characteristics, highlighting the value of more detailed body composition measures, such as muscle mass.

Given the limited evidence, further research is needed to better understand the role of anthropometric characteristics in relation to pain experience in children with cancer.

### 4.4. Implications for Clinical Practice

Despite pain being a primary concern of children with cancer and their parents, current pain management approaches remain insufficient [78,79,80]. Possible contributing factors include parental expectations and interpretations of cancer pain, fear of medication side effects, and lack of proper pain education [11,79]. Additionally, underreporting of pain by children with cancer due to communication difficulties or social restrictions may further complicate adequate pain management [81]. The observed differences in pain between children with cancer and healthy controls underscore the need for tailored, multimodal pain management. These should incorporate pharmacological, physical, and psychosocial interventions, tailored to the child’s treatment phase and reported symptoms. Changes in body composition, such as increased fat % and waist circumference, may negatively impact metabolic health, relapse risk, and survival [28,29,65,82,83,84]. Contributing factors may include initial weight at diagnosis, imbalanced diet, immobilization and physical inactivity during treatment [25,61,85,86]. Since unhealthy weight often starts in the early treatment phase [25,61], early promotion of a healthy lifestyle is recommended [25,61,85,86]. Structured exercise programs during and after treatment may help preserve or increase muscle mass, improve functional capacity, and potentially reduce pain sensitivity. Early, individualized nutritional counseling can prevent excessive fat accumulation and support healthy growth. Routine monitoring of body composition and pain throughout treatment allows timely adjustments in rehabilitation and lifestyle interventions. Together, these strategies can enhance both short- and long-term quality of life in children with cancer.

### 4.5. Limitations and Future Directions

Several limitations should be considered. First, due to the cross-sectional study design, causality could not be examined. Second, PPTs were only assessed at 2 test sites (MTA and MTD). Assessing PPTs at a larger number of anatomical sites, or at least adding one site at the upper limb (e.g., ball of the thumb), could provide a more detailed mapping of pressure pain sensitivity and general hyperalgesia. However, our choice of two test sites was a deliberate methodological decision grounded in both scientific rationale and ethical considerations for pediatric research, minimizing participant burden and reducing procedural discomfort. Third, chronic pain [44] was only parent-reported and reduced to a binary classification (yes/no). Moreover, other pain outcomes only covered a limited timeframe (i.e., during the past 2 weeks or at 1 timepoint for the PPTs), potentially missing important nuances. Future studies should also further explore influences of different diagnoses and treatments, as the limited sample size did not allow for subgroup analyses. Another key consideration is that children with cancer were still undergoing active treatment and may have received varying pain management strategies, possibly biasing their pain reports. While children’s medication use was evaluated, the fact that we could not obtain medication data through the patient records and that this data was exclusively parent-reported (including parents reporting “I don’t know”), in addition to not asking for the intake of analgesics during the day of study participation specifically, may have biased our PPT results. Medication data was also not adjusted for in the statistical analysis. The impact of analgesics (e.g., paracetamol) on PPTs depends on the type of medication, the underlying pain mechanism, and the timing of measurement. In short, most analgesics slightly raise PPTs, although the effect size and duration vary widely. Future studies should control for the use of analgesics to confirm the findings of the current study. Additionally, although exceeding the scope and purpose of the current study, focusing on clinical and physiological measures of pain (sensitivity) and anthropometric characteristics, genetic and pharmacogenomic factors are increasingly recognized as important determinants of cancer pain experience and analgesic response. In the era of precision oncology, individual variability in pain sensitivity and treatment response may be partly explained by genetic polymorphisms influencing nociception, inflammation, and opioid receptor function. Future pediatric oncology studies should therefore integrate genetic testing into pain research protocols, to enable the development of tailored pain management strategies that combine physiological, psychosocial, and molecular profiles for adequate and individualized care. Lastly, given the modest sample size (N = 60), the statistical power of our multivariable regression models may have been limited, and findings should therefore be interpreted with caution and confirmed in larger cohorts.

## 5. Conclusions

In conclusion, this study adds to the evidence that pain characteristics and anthropometric characteristics differ between children with cancer compared to healthy peers and identifies factors associated with pain. Given that both pain and body composition changes can have adverse consequences, the current study findings highlight the need for early, personalized pain management approaches and body composition monitoring in pediatric cancer care and may guide future clinical conversations. Future research should focus on longitudinal designs to better understand the trajectories of pain and body composition throughout treatment and survivorship, as well as interventional studies testing the effectiveness of structured exercise, nutritional counseling, and multimodal pain management strategies in improving both short- and long-term outcomes.

## Figures and Tables

**Table 1:** Participant characteristics.

Characteristic		Healthy Controls (n = 30)	Children with Cancer (n = 30)
Age of children; M (SD)		10.97 (2.40)	11.73 (2.49)
Sex of children; n (%)	Girls Boys	13 (43.33) 17 (56.67)	13 (43.33) 17 (56.67)
Nationality of children; n (%)	Belgium The Netherlands	29 (96.67) 1 (3.33)	30 (100.00) 0 (0.00)
Age of parents; M (SD)		43.67 (5.66)	42.00 (6.06)
Sex of parents; n (%)	Mothers Fathers	23 (76.67) 7 (23.33)	20 (66.67) 10 (33.33)
Self-reported health status of parent; n (%)	Excellent Very good Good Moderate Poor	8 (26.67) 12 (40.00) 10 (33.33) 0 (0.00) 0 (0.00)	2 (6.67) 9 (30.00) 16 (53.33) 3 (10.00) 0 (0.00)
Education level of parent; n (%)	Primary education (≤12 yo) Lower secondary education (≤14 yo) Higher secondary education (≤18 yo) Higher education (bachelor/master)	3 (10.00) 1 (3.33) 3 (10.00) 23 (76.67)	0 (0.00) 3 (10.00) 11 (36.67) 16 (53.33)
Occupation of parent; n (%)	Housewife/househusband Laborer Employee Liberal profession Self-employed Manager position Unemployed/invalidity Official/public servant	0 (0.00) 1 (3.33) 24 (80.00) 1 (3.33) 1 (3.33) 0 (0.00) 0 (0.00) 3 (10.00)	0 (0.00) 6 (20.00) 16 (53.33) 0 (0.00) 3 (10.00) 1 (3.33) 2 (6.67) 2 (6.67)

M = mean; SD = standard deviation; yo = years old.

**Table 2:** Medical characteristics of children with cancer.

Characteristic		n = 30
Diagnosis; n (%)	ALL AML CML Hodgkin lymphoma T-cell lymphoma Germ cell tumor Thyroid carcinoma Rhabdomyosarcoma Medulloblastoma Neuroblastoma Osteosarcoma Ewing sarcoma	12 (40.00) 2 (6.67) 1 (3.33) 4 (13.33) 1 (3.33) 3 (10.00) 1 (3.33) 1 (3.33) 1 (3.33) 1 (3.33) 1 (3.33) 2 (6.67)
Time since diagnosis in months; M (SD)		6.70 (7.49)
Treatment; n (%)	Chemotherapy Chemotherapy/surgery Chemotherapy/radiotherapy Chemotherapy/immunotherapy Chemotherapy/Imatinib Chemotherapy/Imatinib/immunotherapy Chemotherapy/radiotherapy/surgery Chemotherapy/extracorporeal irradiation/surgery Chemotherapy/immunotherapy/surgery/radiotherapy/ASCT Surgery/radiotherapy	17 (56.67) 2 (6.67) 2 (6.67) 1 (3.33) 1 (3.33) 1 (3.33) 3 (10.00) 1 (3.33) 1 (3.33) 1 (3.33)

M = mean; SD = standard deviation ALL = acute lymphocytic leukemia; AML = acute myelocytic leukemia; CML = chronic myelocytic leukemia; ASCT = autologous stem cell transplant.

**Table 3:** Child pain characteristics.

Characteristic		Healthy Controls (n = 30)	Children with Cancer (n = 30)	Median Difference (Hodges-Lehman) (95% CI)	Test Statistic	*p*-Value	Effect Size
Child chronic pain (parent report); n (%) ^a^	Yes No	0 (0.00) 30 (100.00)	7 (23.33) 23 (76.67)			0.011 *	φ = 0.363
Pain in last 2 weeks; n (%) ^a^	Yes No	23 (76.67) 7 (23.33)	16 (53.33) 14 (46.67)		χ^2^ (1) = 3.590	0.058	φ = 0.245
Pain intensity last 2 weeks; n (%) ^a^	No pain Little pain Moderate pain Much pain A whole lot of pain	7 (23.33) 9 (30.00) 9 (30.00) 5 (16.67) 0 (0.00)	14 (46.67) 6 (20.00) 8 (26.67) 1 (3.33) 1 (3.33)			0.138	V = 0.333
Number of pain locations last 2 weeks; Median [IQR] ^b^		1.00 [0.75–2.00]	1.00 [0.00–1.00]	0.00 [0.00–1.00]	U = 318.000	0.037 *	r = −0.269
Pain frequency last 2 weeks; n (%) ^a^	Never Once Several times Often Constantly	7 (23.33) 3 (10.00) 13 (43.33) 6 (20.00) 1 (3.33)	14 (46.67) 3 (10.00) 10 (33.33) 1 (3.33) 2 (6.67)			0.143	V = 0.332
PPT MTA; Median [IQR] ^b^		3.73 [1.91–4.43]	2.05 [1.25–3.81]	1.03 [0.12–1.98]	U = 303.500	0.030 *	r = −0.280
PPT MTD; Median [IQR] ^b^		1.47 [1.00–2.04]	1.18 [0.51–1.96]	0.32 [−0.09–0.70]	U = 350.500	0.141	r = −0.190

The distribution of the continuous data within each group was assessed by histograms, QQ-plots, and the Shapiro–Wilk test. ^a^ Categorical data were analyzed by performing Fisher’s Exact test or Chi-square test as appropriate. Effect sizes were presented as Phi or Cramér’s V for 2 × 2 or larger tables, respectively. ^b^ Continuous data that were not normally distributed and subsequent group differences were analyzed using the Mann–Whitney U Test. Differences are reported as Hodges-Lehman median differences (95% CI), and rank-biserial correlation r as the effect size. Rank-biserial correlations are signed based on the direction of group medians. CI = confidence interval; IQR = interquartile range; PPT = pressure pain threshold; MTA = dominant musculus tibialis anterior; MTD = dominant musculus trapezius descendens. * *p* < 0.05.

**Table 4:** Child anthropometric characteristics.

Characteristic	Healthy Controls (n = 30)	Children with Cancer (n = 30)	Mean/Median Difference (95% CI)	Test Statistic	*p*-Value	Effect Size
Waist circumference (cm); M(SD) ^a^	61.12 (6.60)	67.27 (10.82)	−6.15 [−10.87–−1.44]	t(46.0) = −2.627	0.012 *	d = −0.689
Fat (%); Median [IQR] ^b^	18.35 [16.58–19.98]	20.60 [17.23–24.95]	−2.85 [−5.50–−0.50]	U = 299.000	0.026 *	r = 0.288
Fat-free mass (kg); Median [IQR] ^b^	27.65 [22.90–39.45]	33.70 [23.55–38.93]	−2.30 [−8.20–2.80]	U = 390.500	0.379	r = 0.114
Muscle mass (kg); Median [IQR] ^b^	26.15 [21.88–37.43]	31.95 [22.33–36.90]	−2.15 [−7.80–2.80]	U = 396.500	0.429	r = 0.102
Total body water (%); Median [IQR] ^b^	59.70 [58.40–61.00]	58.15 [55.15–60.40]	2.10 [0.30–3.90]	U = 293.000	0.020 *	r = −0.300
BMI (kg/m^2^); Median [IQR] ^b^	16.90 [14.93–18.73]	16.85 [15.70–19.58]	−0.60 [−2.10–0.90]	U = 396.000	0.425	r = 0.103

The distribution of the continuous data within each group was assessed by histograms, QQ-plots, and the Shapiro–Wilk test. ^a^ Continuous data that were assumed to be normally distributed were analyzed with the independent samples *t*-test. Differences were reported as mean differences (95% CI) and Cohen’s d as the effect size. ^b^ Continuous data that were not normally distributed and subsequent group differences were analyzed using the Mann–Whitney U Test. Differences were reported as Hodges-Lehman median differences (95% CI) and rank-biserial correlation r as the effect size. Rank-biserial correlations are signed based on direction of group medians. CI = confidence interval; M = mean; SD, standard deviation; IQR = interquartile range; BMI = body mass index. * *p* < 0.05; ~n = 29 as one child’s waist circumference could not be measured due to a catheter attached to the abdomen at the measurement site.

**Table 5:** Regression analyses.

	B	Wald χ^2^	Exp (B)	95% CI for Exp (B)	*p*-Value
**Number of pain locations last 2 weeks ^a^**					
(Intercept)	−0.89	0.56	0.41	[0.04–4.18]	0.454
Group (healthy controls vs. children with cancer)	0.52	1.72	1.68	[0.77–3.64]	0.190
Fat (%)	−0.09	1.85	0.91	[0.80–1.04]	0.174
Muscle mass (kg)	0.01	0.12	1.01	[0.94–1.09]	0.725
BMI (kg/m^2^)	0.12	0.69	1.13	[0.84–1.52]	0.407
	**B**	**SE B**	**β**	**t**	***p*** **-value**
**PPT MTA ^b^**					
(Constant)	2.04	0.73		2.80	0.007 *
Group (healthy controls vs. children with cancer)	−1.04	0.42	−0.305	−2.49	0.016 *
Muscle mass (kg)	0.05	0.02	0.267	2.18	0.033 *
**PPT MTD ^c^**					
(Constant)	−0.36	0.27		−1.34	0.185
Group (healthy controls vs. children with cancer)	−0.37	0.16	−0.286	−2.40	0.020 *
Muscle mass (kg)	0.03	0.01	0.361	3.03	0.004 *

^a^ Analyzed via Negative Binominal analysis. The Negative Binominal model was statistically significant, χ^2^(4) = 11.218, *p* = 0.024, indicating that the predictors, as a set, were related to the number of painful locations experienced over the past two weeks. ^b^ Analyzed via multiple regression analysis, using a stepwise approach. The final model was statistically significant, F(2, 57) = 5.08, *p* = 0.009, explaining 15.1% (R^2^ = 0.151, adjusted R^2^ = 0.121) of the variance in the PPT of the musculus tibialis anterior. ^c^ Analyzed via multiple regression analysis, the predictors were entered simultaneously. The final multiple regression model was statistically significant, F(2, 57) = 6.9, *p* = 0.002, explaining 19.5% of the variance (R^2^ = 0.195, adjusted R^2^ = 0.167) in the log-transformed PPT of the musculus trapezius descendens. SE = standard error; BMI = body mass index; PPT = pressure pain threshold; MTA = dominant musculus tibialis anterior; MTD = dominant musculus trapezius descendens. * *p* < 0.05.

## Data Availability

The data that support the findings of this study are available upon reasonable request from the corresponding author. The data are not publicly available due to privacy and ethical restrictions.

## Supplementary Materials

The following supporting information can be downloaded at: https://www.mdpi.com/article/10.3390/children12091166/s1, Table S1: Exact percentages of painful locations reported by children who experienced pain in the past 2 weeks, corresponding to the heat map in Figure 1. The first column lists the body locations; the second column shows the percentages for the healthy controls of those who reported pain in the past 2 weeks (Figure 1a), and the third column shows the percentages for the children with cancer of those who reported pain in the past 2 weeks (Figure 1b).

## Abbreviations

The following abbreviations are used in this manuscript:

BMI	Body Mass Index
MTA	Musculus tibialis anterior
MTD	Musculus trapezius pars descendens
PPT	Pressure pain threshold
QST	Quantitative Sensory Testing
