# Supplementary material for: Exploring Pain and Body Composition in Children with Cancer Compared to Healthy Controls: A Cross-Sectional Case-Control Study
Source: Children (Basel). 2025 Sep 1;12(9):1166. doi: 10.3390/children12091166 (PMC12468649; doi:10.3390/children12091166)
Supplement: Supplementary file 1 [file children-12-01166-s001.zip › children-3751781-supplementary.pdf]

Table S1. Exact percentages of painful locations reported by children who experienced pain in the past 2 weeks, corresponding to the heat map in *Figure 1*.

**Location = code**	**Figure 1 - Panel (a)**<br>**N(%) healthy controls of those who reported pain in the past 2 weeks (*n*=23)**	**Figure 1 – Panel (b)**<br>**N(%) children with cancer of those who reported pain in the past 2 weeks (*n*=16)**
Shoulder girdle left = 0	2 (8,70%)	1 (6,25%)
Shoulder girdle right = 1	1 (4,35%)	0 (0,00%)
Upper arm left = 2	2 (8,70%)	0 (0,00%)
Upper arm right = 3	3 (13,04%)	0 (0,00%)
Forearm left = 4	3 (13,04%)	0 (0,00%)
Forearm right = 5	2 (8,70%)	0 (0,00%)
Hip (buttock) left = 6	0 (0,00%)	0 (0,00%)
Hip (buttock) right = 7	1 (4,35%)	1 (6,25%)
Thigh left = 8	4 (17,39%)	2 (12,5%)
Thigh right = 9	3 (13,04%)	1 (6,25%)
Lower leg left = 10	1 (4,35%)	1 (6,25%)
Lower leg right = 11	3 (13,04%)	0 (0,00%)
Jaw left = 12	0 (0,00%)	2 (12,5%)
Jaw right = 13	0 (0,00%)	3 (18,75%)
Chest = 14	2 (8,70%)	1 (6,25%)
Abdomen = 15	7 (30,43%)	3 (18,75%)
Throat/Neck = 16	4 (17,39%) neck	2 neck (12,5%); 1 throat (6,25%)
Upper back = 17	2 (8,70%)	0 (0,00%)
Lower back = 18	2 (8,70%)	1 (6,25%)
Knees = 19	4 (17,39%)	3 (18,75%)
Head = 20	4 (17,39%)	2 (12,5%)
Feet = 21	3 (13,04%)	3 (18,75%)
